# Modifications of Mitochondrial Network Morphology Affect the MAVS-Dependent Immune Response in L929 Murine Fibroblasts during Ectromelia Virus Infection

**DOI:** 10.3390/pathogens13090717

**Published:** 2024-08-23

**Authors:** Karolina Gregorczyk-Zboroch, Lidia Szulc-Dąbrowska, Pola Pruchniak, Małgorzata Gieryńska, Matylda Barbara Mielcarska, Zuzanna Biernacka, Zbigniew Wyżewski, Iwona Lasocka, Weronika Świtlik, Alicja Szepietowska, Patrycja Kukier, Aleksandra Kwiecień-Dębska, Jakub Kłęk

**Affiliations:** 1 Division of Immunology, Department of Preclinical Sciences, Faculty of Veterinary Medicine, Warsaw University of Life Sciences, 02-786 Warsaw, Poland; 2 Institute of Biological Sciences, Cardinal Stefan Wyszyński University in Warsaw, 01-815 Warsaw, Poland; 3 Department of Biology of Animal Environment, Institute of Animal Science, Warsaw University of Life Sciences, 02-786 Warsaw, Poland; 4 Department of Biochemistry and Microbiology, Institute of Biology, Warsaw University of Life Sciences, 02-776 Warsaw, Poland

**Keywords:** MAVS, ECTV, mitochondrial network, immunity, mitochondrial dynamics, poxviruses

## Abstract

Since smallpox vaccination was discontinued in 1980, there has been a resurgence of poxvirus infections, particularly the monkeypox virus. Without a global recommendation to use the smallpox vaccine, the population is not immune, posing a severe threat to public health. Given these circumstances, it is crucial to understand the relationship between poxviruses and their hosts. Therefore, this study focuses on the ectromelia virus, the causative agent of mousepox, which serves as an excellent model for studying poxvirus pathogenesis. Additionally, we investigated the role of mitochondria in innate antiviral immunity during ECTV infection, focusing specifically on mitochondrial antiviral signaling protein. The study used a Moscow strain of ECTV and L929 mouse fibroblasts. Cells were treated with ECTV and chemical modulators of mitochondrial network: Mdivi-1 and CCCP. Our investigation revealed that an elongated mitochondrial network attenuates the suppression of MAVS-dependent immunity by ECTV and reduces ECTV replication in L929 fibroblasts compared to cells with an unaltered mitochondrial network. Conversely, a fragmented mitochondrial network reduces the number of progeny virions while increasing the inhibition of the virus-induced immune response during infection. In conclusion, our study showed that modifications of mitochondrial network morphology alter MAVS-dependent immunity in ECTV-infected mouse L929 fibroblasts.

## 1. Introduction

Poxvirus infections have been noted with increasing frequency over the past years [1,2]. The Centers for Disease Control and Prevention (CDC) reported the detection of an unprecedented global outbreak of monkeypox (mpox) in May 2022, which caused significant public health concerns [3]. The mentioned zoonotic disease is caused by the monkeypox virus (MPXV) that belongs to the *Poxviridae* family and *Orthopoxvirus* genus, together with the variola virus (VARV)—the agent responsible for smallpox. Both pathogens can infect humans, and even though smallpox was more threatening, mpox also contributes to developing severe symptoms [2]. Poxviral zoonosis can also be caused by infection with the cowpox virus (CPXV) and vaccinia virus (VACV). Together with mpox, the diseases they cause have been reported in Africa, Europe, India, North America, and South America [4,5]. It is important to note that vaccination against VARV was discontinued after the successful eradication of smallpox in 1980 by the World Health Organization (WHO) [6]. Currently, there are no recommendations for routine vaccination of the general public with the smallpox vaccine. This means that the global population no longer has immunity to not only VARV but also to other poxviruses, e.g., MPXV. Moreover, smallpox remains a potential bioterrorism threat, mainly due to its eradication and the cessation of vaccination efforts. Taken together, researching the poxvirus–host relationship is entirely justified and necessary. Therefore, our work focused on the ectromelia virus (ECTV), the causative agent of mousepox, for several reasons: (1) Infection with this pathogen primarily affects susceptible strains of mice but not humans—it is safe in handling and containment; (2) the disease progression and pathology of mousepox closely resemble those of human smallpox, making ECTV an excellent model for studying the pathogenesis of poxvirus infections; and (3) ECTV shares genetic similarities with other poxviruses—it can provide insights into the mechanisms of poxvirus replication, host interactions, and immune evasion strategies.

Besides the ECTV, our work also involved mitochondria. As we have known for years, they play a pivotal role in innate antiviral immunity [7,8]. Mitochondria serve as a signaling platform to produce proinflammatory cytokines and type I interferon (IFN) owing to mitochondrial antiviral-signaling protein (MAVS), also known as IPS1 (IFN-β promoter stimulator I), Cardif (caspase activation recruitment domain adaptor inducing IFN-β), and VISA (virus-induced signaling adaptor) [9]. Castanier and colleagues (2010) have suggested that the mitochondrial network dynamics affect MAVS-dependent immunity. Elongation of this structure increases signaling downstream from MAVS, whereas fragmentation presents the opposite effect [10]. Based on these assumptions, one might suspect that viral infections lead to mitochondrial network fission to evade immune mechanisms. However, looking at the significant mitochondrial function, i.e., providing energy, this hypothesis is no longer so obvious. Numerous studies have shown that excessive mitochondrial fragmentation may be associated with lower levels of ATP, cellular dysfunction, and disease states that are not conducive to viral infection development [11,12]. Moreover, mitochondrial dysfunction related to ATP depletion and reactive oxygen species (ROS) production may lead to apoptosis or autophagy, which can either limit or intensify viral replication depending on the particular virus type [13]. Consequently, it remains a mystery which strategy will be more beneficial to the virus.

As demonstrated in our previous work, ECTV infection leads to the mitochondrial network reorganization and fragmentation in the murine L929 fibroblasts and RAW 264.7 macrophages [14,15]. Therefore, we aimed to investigate the association between the state of mitochondrial network morphology and MAVS-dependent immunity in ECTV-infected cells. We found that an elongated mitochondrial network reduces the impact of ECTV infection on MAVS-dependent immunity inhibition and decreases the ECTV replication level in L929 murine fibroblasts compared to cells with the physiological mitochondrial network. Going further, a fragmented mitochondrial network also reduces the number of progeny virions but intensifies virus-mediated braking of the immune response during infection. With these things taken together, our study showed the impact of mitochondrial network morphology manipulations on MAVS-dependent immunity in L929 murine fibroblasts infected with ECTV.

## 2. Materials and Methods

### 2.1. Virus

All experiments used a highly virulent Moscow strain of ectromelia virus (ECTV-MOS; VR1374; American Type Culture Collection (ATCC), Manassas, VA, USA). ECTV-MOS propagation and titration were performed in the Vero monkey cell line (CCL-81) obtained from ATCC, which was purified and stored at −80 °C until use.

### 2.2. Cell Lines

The L929 murine fibroblast cell line (CCL-1), obtained from ATCC (Manassas, VA, USA), was used as the main cell model in the present study. Cells were cultured in a growth medium referred to as normal, which contained DMEM with 4.5 g/L glucose and 4.0 mM L glutamine supplemented with 5% fetal bovine serum (FBS) and 1% penicillin–streptomycin–amphotericin B solution (Sigma-Aldrich, St. Louis, MO, USA) at 37 °C, with 5% CO_2_ in a humidified incubator.

Some studies were performed on the RAW 264.7 murine macrophage cell line (TIB-71) obtained from ATCC (Manassas, VA, USA). They were cultured in RPMI 1640-GlutaMAX medium (Gibco, Waltham, MA, USA) supplemented with 10% FBS and 1% antibiotic solution containing 100 U/mL penicillin and 100 μg/mL streptomycin (Sigma-Aldrich, Saint Louis, MO, USA) at 37 °C, with 5% CO_2_ in a humidified incubator.

### 2.3. Chemicals, siRNAs and Antibodies

DMSO (di-methylsulfoxide; Sigma-Aldrich, St. Louis, MO, USA) was used as a solvent for other chemicals, and DMSO-treated cells were considered as a control; Mdivi-1 (mitochondrial division inhibitor 1; Sigma-Aldrich, St. Louis, MO, USA) is a selective inhibitor of Drp1 (dynamin-related protein 1) used for mitochondrial network elongation; CCCP (carbonyl cyanide m-chlorophenyl hydrazone; Sigma-Aldrich, St. Louis, MO, USA) is a chemical inhibitor of oxidative phosphorylation that decreases mitochondrial membrane potential, leading to mitochondrial fragmentation; poly(I:C) (HMW)/LyoVec (Invivogen, San Diego, CA, USA) is a synthetic dsRNA polymer with the transfection reagent LyoVec used as RIG-I/MDA-5 signaling pathway activator; Mito Red (Sigma-Aldrich, St. Louis, MO, USA) is a red fluorescent probe used for labeling mitochondria in living cells; Hoechst 33342 is a blue fluorescent probe labeling cellular and foreign DNA. Mfn1 (Mitofusin 1) siRNA (mouse specific) (Santa Cruz Biotechnology, Dallas, TX, USA); Drp1 (Dynamin related protein 1) siRNA (mouse specific) (Santa Cruz Biotechnology, Dallas, TX, USA); control siRNA-A (Santa Cruz Biotechnology, Dallas, TX, USA); and siRNA Transfection Reagent (Santa Cruz Biotechnology, Dallas, TX, USA) were also used. Primary antibodies used in the study are listed in Table 1. Secondary anti-mouse or anti-rabbit pAb conjugated to fluorescein isothiocyanate (FITC) or Rhodamine Red-X (Jackson ImmunoResearch Laboratories, West Grove, PA, USA) and secondary anti-mouse or anti-rabbit pAb conjugated to horseradish peroxidase (HRP) (Cell Signaling Technology, Danvers, MA, USA) were used.

### 2.4. Measurement of Cell Toxicity of DMSO, Mdivi-1, and CCCP

A trypan blue exclusion test evaluated DMSO, Mdivi-1, and CCCP cell toxicity. The experiments were performed 6, 13, 19, or 25 h post treatment. Cells were detached with 0.25% trypsin-EDTA (Thermo Fisher Scientific, Waltham, MA, USA), mixed with 0.4% trypan blue (Thermo Fisher Scientific, Waltham, MA, USA) in a ratio 1:1 with the cell suspension and counted using a Neubauer chamber. Only dead cells were trypan blue-positive, while living cells with no destroyed cell membranes remained unlabeled. The percentage of dead cells provided insight into the cell toxicity of the selected chemicals.

### 2.5. ECTV Infection Procedure

L929 or RAW 264.7 cells were cultured either in a 6-well culture plate or on round coverslips in a 24-well culture plate using an appropriate medium. The media were replaced with new ones containing a 5-fold lower serum concentration for the infection procedure. Subsequently, the cells were treated with either DMSO, Mdivi-1 (50 µM for L929 cells; 20 µM for RAW 264.7 cells), or CCCP (20 µM for L929 cells; 5 µM for RAW 264.7 cells) and transfected or not with 1 mg/mL poly(I:C) (HMW) LyoVec. Following this, the cells were infected with ECTV at a multiplicity of infection (MOI) of 1 or 5. Throughout the 1 h virus adsorption period at 37 °C and 5% CO_2_, the plates were rocked every 15 min to enhance virus–cell contact.

### 2.6. Immunofluorescent Staining

At the indicated time of infection, fibroblasts or macrophages were fixed with a 4% paraformaldehyde (PFA) solution, permeabilized with a 0.5% Triton X-100 (Sigma-Aldrich, St. Louis, MO, USA) solution in PBS for 10 min, and blocked with 3% bovine serum albumin (BSA) (Sigma-Aldrich, St. Louis, MO, USA) in 0.1% Triton X-100 in PBS for 30 min. Slides were then incubated with protein target-specific unlabeled antibodies for 1 h at room temperature (RT).

After washing with 0.1% Triton X-100 in PBS, primary antibodies were detected by FITC/Rhodamine Red-X-conjugated secondary antibodies. Slides were then mounted in Duolink in Situ Mounting Medium with DAPI (Sigma-Aldrich, St. Louis, MO, USA). Images were captured using an Olympus BX60 fluorescence microscope (Olympus, Tokyo, Japan) equipped with a PROMICAM 3-5CP camera and QuickPHOTO 2.3 software (Promicra, Prague, Czech Republic). Image analysis was performed using CellˆF 1.2 software (Olympus, Tokyo, Japan) and ImageJ 1.48v software (NIH, Bethesda, MD, USA).

### 2.7. Proximity Ligation Assay

Protein–protein closeness was detected using the proximity ligation assay (PLA). It relies on recognizing and amplifying signals generated when two proteins of interest are near (≤40 nm) each other. PLA was employed to visualize the co-localization of MAVS with other proteins involved in (1) recognition of viral infection (RIG-I, MDA-5, and STING) and (2) mitochondrial network dynamics (Mfn1, Mfn2, Opa1, Drp1, Fis1, and Mff).

Cell preparation for the PLA technique followed the procedure described in Section 4 up to the blocking step. After the blocking step, L929 fibroblasts were incubated with primary unlabeled antibodies against MAVS and one of the following: RIG-I, MDA-5, STING, Mfn1, Mfn2, Opa1, Drp1, Fis1, or Mff for 1 h at 37 °C in a humidity chamber. Slides were then washed with Tris-buffered saline supplemented with 0.05% Tween 20 (TBS-T) and incubated with anti-mouse and anti-rabbit oligonucleotide-conjugated secondary antibodies (Navenibody), supplied by the producer of the NaveniFlex Cell MR kit (Navinci Diagnostics, Uppsala, Sweden), in a preheated humidity chamber for 1 h at 37 °C.

After washing with prewarmed TBS-T, cells were incubated with enzyme 1 for 30 min at 37 °C in a humidity chamber. If the two probes were in close proximity due to the interaction of their target proteins, the DNA oligonucleotides attached to them could hybridize to form a circular DNA molecule. Slides were then washed with TBS-T and incubated with enzyme 2 in a preheated humidity chamber for 1.5 h at 37 °C. DNA polymerase was used to amplify the circular DNA molecules through a rolling circle amplification (RCA). The amplified DNA products were visualized using fluorescence-labeled probe. The red fluorescence signal indicated the presence of the protein–protein closeness. 

After the washing step with TBS, slides were mounted in Duolink in Situ Mounting Medium with DAPI (Sigma-Aldrich, St. Louis, MO, USA). Data were collected using an Olympus BX60 fluorescence microscope (Olympus, Tokyo, Japan) equipped with a PROMICAM 3-5CP camera and QuickPHOTO 2.3 software (Promicra, Prague, Czech Republic). Image analysis was performed using CellˆF 1.2 software (Olympus, Tokyo, Japan) and ImageJ 1.48v software (NIH, Bethesda, MD, USA).

### 2.8. Flow Cytometry

L929 cells were seeded in a 6-well culture plate at a density of 2 × 10^5^ cells per well and treated as described in Section 4. Subsequently, cells were detached with 0.25% trypsin-EDTA and centrifuged (300× *g*, RT, 5 min). After two washings with cold PBS and centrifugation, the cells were fixed and permeabilized using BD Cytofix/Cytoperm Fixation/Permeabilization Kit (Becton Dickinson and Company, San Jose, CA, USA). The fibroblasts were then transferred to a 96-well plate and labeled with anti-MAVS primary antibodies (30 min on ice). Following washing steps, secondary anti-mouse APC-labeled antibodies were applied (30 min on ice). The prepared cell suspension was analyzed using BD LSRFortessa Cell Analyzer (Becton Dickinson and Company, San Jose, CA, USA), and the acquired data were analyzed using BD FACSDiva 7.0 software (Becton Dickinson and Company, San Jose, CA, USA). A total of 2 × 10^4^ events were collected for each sample, and data were presented as mean fluorescent intensity (MFI).

### 2.9. Western Blot

L929 cells were seeded in a 6-well culture plate at a density of 2 × 10^5^ cells per well and treated as described in Section 4. At 24 h post infection (hpi) with ECTV, proteins were extracted from cells using RIPA buffer (Thermo Fisher Scientific, Waltham, MA, USA) supplemented with a 1% protease/phosphatase inhibitor cocktail (Thermo Fisher Scientific, Waltham, MA, USA). Additionally, the post-culture cell medium was used in ELISA as described in Section 2.11. The protein concentration was determined using the BCA assay (Thermo Fisher Scientific, Waltham, MA, USA) and an Epoch BioTek spectrophotometer (BioTek Instruments, Inc., Winooski, VT, USA). The obtained data were analyzed using Gen5 software version 3.11 (BioTek Instruments, Inc., Winooski, VT, USA). The extracted proteins were electrophoretically separated based on their molecular size on 4–12% gradient polyacrylamide concentration Bolt Bis-Tris Plus gels (Thermo Fisher Scientific, Waltham, MA, USA) using a Bolt Mini Gel Tank (Thermo Fisher Scientific, Waltham, MA, USA). After electrophoresis, the separated proteins were transferred onto a solid PVDF (polyvinylidene difluoride) membrane. The membrane was then incubated in a blocking solution (non-fat milk or BSA) and probed with a specific primary antibody that binds to the target protein of interest (MAVS, STING, MDA-5, RIG-I, pIRF3, GAPDH, Mfn1, and Mfn2). The primary antibody was diluted in a blocking buffer and incubated with the membrane overnight at 4 °C. After washing, the membrane was then incubated with a secondary antibody conjugated to an enzyme (HRP) and then washed and treated with SuperSignal West Pico PLUS Chemiluminescent Substrate (Thermo Fisher Scientific, Waltham, MA, USA). The resulting signal was visualized using X-ray films. The intensity of the protein bands on the membrane was quantified using ImageJ 1.48v software (NIH, Bethesda, MD, USA), and the relative expression levels of the target protein were determined by comparing them to GAPDH.

### 2.10. siRNA Transfection

For the siRNA transfection procedure, L929 cells were seeded in a 6-well culture plate at a density of 2 × 10^5^ cells per well in an antibiotic-free growth medium. The cells were then incubated at 37 °C in a 5% CO_2_ atmosphere until they reached 60–80% confluence. Subsequently, they were treated with a transfection reagent mixture containing 10 µM of the appropriate siRNA (control, Drp1, or Mfn1), Transfection Reagent, and Transfection Medium (Santa Cruz Biotechnology, Dallas, TX, USA). After a 7 h incubation period at 37 °C with 5% CO_2_, the normal growth medium, containing twice the normal serum and antibiotics concentration, was added without removing the transfection mixture. The cells were then further incubated for an additional 24 h, after which the growth medium was replaced with a fresh, normal cell culture medium. At 48 or 72 h after the addition of the transfection reagent mixture, the cells were infected with ECTV as described in Section 4. The success of transfection was verified using the Western blot technique.

### 2.11. Immunoenzyme Assay

L929 cells were seeded in a 6-well culture plate and treated as described in Section 4. Quantitative measurement of IFN type I (α and β) and proinflammatory cytokines (IL-1β, IL-6, and TNFα) levels in post-cell culture media was performed using indirect enzyme-linked immunosorbent assays (ELISA) (Thermo Fisher Scientific, Waltham, MA, USA) according to the manufacturer’s protocols. The absorbance in each well was measured using a microplate reader and proportional to the amount of antibodies in the sample. The results were analyzed by comparing the absorbance values of the samples with those of known standards. The antibody titer or concentration was determined in the samples based on the standard curve.

### 2.12. Plaque Assay

Vero cells were cultured in 24-well plates until they reached confluence to perform the plaque assay. Subsequently, the cells were infected with 10-fold serial dilutions of ECTV suspension obtained from L929 or RAW 264.7 cells treated with chemicals (DMSO, Mdivi-1 or CCCP) or transfected with siRNA (control siRNA, Drp1 siRNA, or Mfn1 siRNA) at 6, 10, or 18 hpi. At 4 days post infection, Vero cells were fixed in 4% PFA stained with 0.3% crystal violet and air dried. The formed plaques of ECTV were then counted using an Olympus IX71 inverted microscope (Olympus, Tokyo, Japan).

### 2.13. Statistical Analysis

Results are presented as mean ± standard deviation (SD) or median and interquartile range (IQR) from three independent biological replicates. Statistical analysis was performed using STATISTICA 13.0 software (StatSoft Inc., Tulsa, OK, USA) and Microsoft Excel 2019 Microsoft Office (Microsoft, Redmond, WA, USA) using paired Student’s *t*-test or Wilcoxon signed-rank test. Statistical significance was evaluated at * *p* ≤ 0.05, ** *p* ≤ 0.01, and *** *p* ≤ 0.001.

### 2.14. Language Correction

Language proofreading was performed using ChatGPT 3.5 (Open AI, San Francisco, CA, USA; https://chatgpt.com) and Grammarly (San Francisco, CA, USA). 

## 3. Results

### 3.1. Modifiers of Mitochondrial Network Morphology Exhibit Varying Degrees of Cell Toxicity between L929 and RAW 264.7

Our studies revealed that DMSO exhibited minimal cell toxicity compared to both untreated L929 and RAW 264.7 cells (~1.3% and ~2.6%, respectively) after 25 h (1 h of virus adsorption + 24 h) of incubation. In L929 fibroblasts, treatment with 50 µM Mdivi-1 resulted in an average toxicity of 5.3% after a 7 h (1 h of virus adsorption + 6 h) incubation period. However, in RAW 264.7 macrophages, the average toxicity was notably higher, reaching 60.1% during the same timeframe. Consequently, the dose of Mdivi-1 was reduced to 20 µM in RAW 264.7 cells, yet significant toxicity of 20.1% persisted after 7 h of incubation. Prolonged incubation periods (13 or 19 h) led to even greater cell death rates, with at least 73.8% observed after 19 h (1 h of virus adsorption + 18 h), prompting us to select 7 h as the maximum incubation time. In L929 fibroblasts, approximately 15.6% cell toxicity was noted after 19 h and 23.6% after 25 h of incubation with 50 µM Mdivi-1. Based on these results the 19 h timeframe was chosen for most of the experiments.

Furthermore, it was found that either 50 µM or 20 µM Mdivi-1 concentration was minimal to achieve elongated mitochondrial networks in L929 cells or RAW 264.7 cells, respectively.

Additionally, 20 µM CCCP in L929 fibroblasts or 5 µM in RAW 264.7 macrophages were the minimal concentrations required to obtain a complete fragmented mitochondrial network. A ~13.9% or 28.4% cell toxicity was observed after 7 h in L929 and RAW 264.7 cells, respectively. Toxicity was raised to ~18.9% after 19 h in L929 cells and to 80.3% in RAW 264.7 cells. At 25 h post treatment, an average of 27.7% cell death was observed in L929 fibroblasts and 97.1% in RAW 264.7 macrophages.

In summary, RAW 264.7 macrophages exhibited greater sensitivity to Mdivi-1 and CCCP treatments than L929 fibroblasts.

### 3.2. The State of Mitochondrial Network Morphology Affects the Level of ECTV-MOS Replication

**Figure 1:**
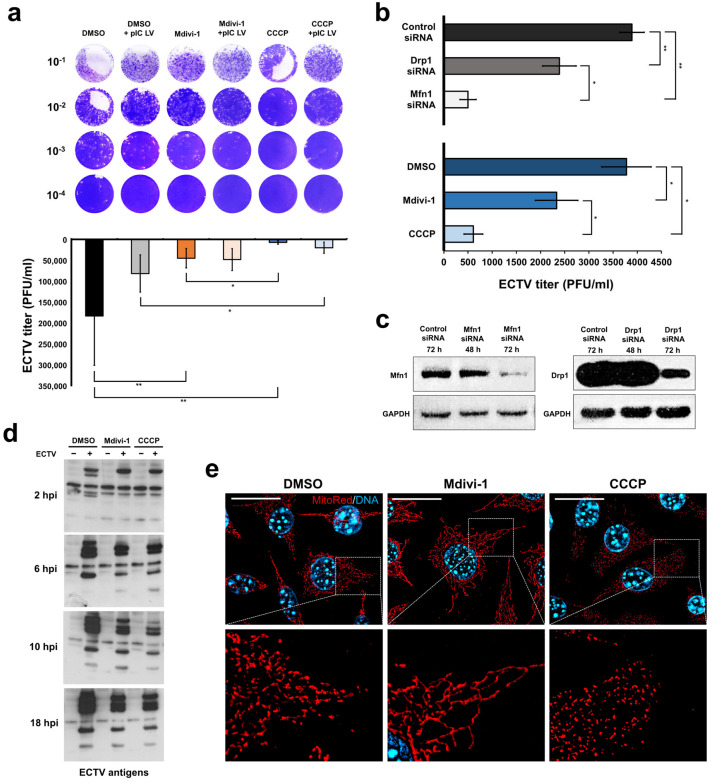
Ectromelia virus (ECTV) replication in L929 murine fibroblasts with modified mitochondrial network morphology. The plaque assay determined the ECTV titer in L929 cells at two time points: (**a**) 18 h post infection (hpi) under various treatments, including DMSO, Mdivi-1, or CCCP, with or without transfection of poly(I:C) (MHW) LyoVec (pIC LV); and (**b**) 10 hpi with transfection of control siRNA, Mfn1 siRNA, or Drp1 siRNA or treatment with DMSO, Mdivi-1, or CCCP. The data from both (**a**,**b**) are presented as mean ± standard deviation (SD), with statistical significance indicated as * *p* ≤ 0.05 and ** *p* ≤ 0.01. ECTV suspension was obtained from cell lysates in post-culture media. Western blot analysis was performed to confirm Mfn1 or Drp1 gene silencing in target cells (**c**) and to assess ECTV antigens expression in control and infected cells treated with DMSO, Mdivi-1, or CCCP at 2, 6, 10, and 18 hpi (**d**). (**e**) Fluorescence verification of mitochondrial network morphology in L929 fibroblast after treatment with DMSO (intact network), Mdivi-1 (elongated), and CCCP (fragmented) by using MitoRed fluorochrome (red). Scale bar: 20 µm.

Studies conducted with mitochondrial network modifiers (Mdivi-1 or CCCP) or siRNA transfection to silence genes encoding proteins involved in mitochondrial fusion (Mfn1 siRNA) and fragmentation (Drp1 siRNA) revealed that the highest level of ECTV-MOS replication (10 hpi or 18 hpi) occurred in L929 fibroblasts with an intact mitochondrial network (treated with DMSO or control siRNA) (Figure 1). In contrast, the lowest number of progeny virions was observed in cells with a fragmented network. Disruption of mitochondrial dynamics, either by excessive fragmentation or elongation of the mitochondria, reduces the level of ECTV replication in cells treated with Mdivi-1, CCCP, Mfn1 siRNA, or Drp1 siRNA. The above results indicate that only undisturbed mitochondrial dynamics allow ECTV-MOS replication to be carried out efficiently in L929 cells. 

**Figure 2:**
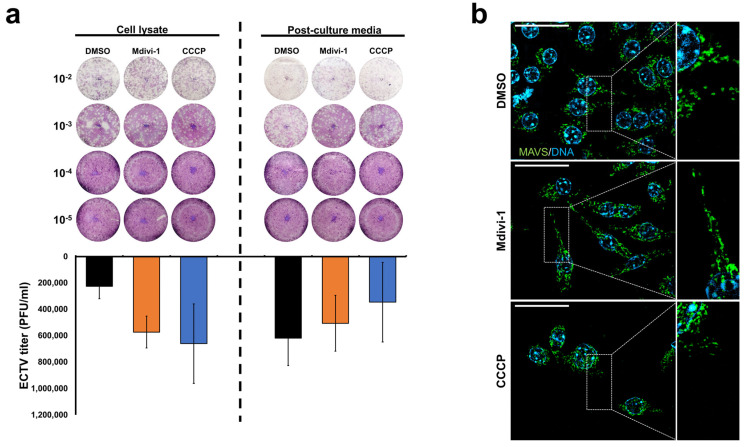
ECTV replication in RAW 264.7 murine macrophages with modified mitochondrial network morphology. (**a**) Determination of ECTV titer by plaque assay in RAW 264.7 cells at 6 hpi treated with DMSO, Mdivi-1, or CCCP. ECTV suspension was obtained from cell lysates or post-culture media. (**b**) Fluorescence verification of mitochondrial network morphology in RAW 264.7 murine macrophages after treatment with DMSO (intact network), Mdivi-1 (elongated), and CCCP (fragmented). Macrophages were labeled using antibodies against MAVS (green) and DNA (blue). Scale bar: 20 µm.

In contrast, studies conducted on RAW 264.7 mouse macrophages infected with ECTV and treated with mitochondrial network modifiers showed no statistically significant differences in the number of progeny virions between cells with unaltered, elongated, and fragmented networks (Figure 2). This lack of significance could be attributed to considerable deviations observed in the results. These findings suggest that the mitochondrial network morphology plays a limited role during ECTV infection in mouse macrophages. This discrepancy likely stems from the distinct functions of these cells, as RAW 264.7 cell mitochondria do not exhibit the elongated and branched structures seen in fibroblasts.

However, when comparing the means of each variant, RAW 264.7 cells treated with CCCP showed the highest virus titer (6.6 × 10^5^ PFU/mL) in cell lysates, whereas those in the DMSO control exhibited the lowest (2.3 × 10^5^ PFU/mL). Interestingly, entirely different results were observed in post-culture media: DMSO treatment yielded the highest number of progeny virions (6.2 × 10^5^ PFU/mL), while CCCP treatment resulted in the lowest (3.5 × 10^5^ PFU/mL). Mdivi-1 usage in both types of ECTV suspensions was similar (5.7 and 5.1 × 10^5^ PFU/mL, respectively). These findings suggest that maintaining an unaltered mitochondrial network morphology may lead to the accelerated release of progeny virions compared to manipulation with modifiers. Furthermore, an utterly fragmented network might hinder the release of virions from the cell.

Since statistically significant results are lacking, firm conclusions cannot be drawn, highlighting the need for further research.

### 3.3. ECTV Infection Alters the Distribution of MAVS Protein in L929 Fibroblasts but Does Not Significantly Impact Its Level in Either L929 or RAW 264.7 Cells

**Figure 3:**
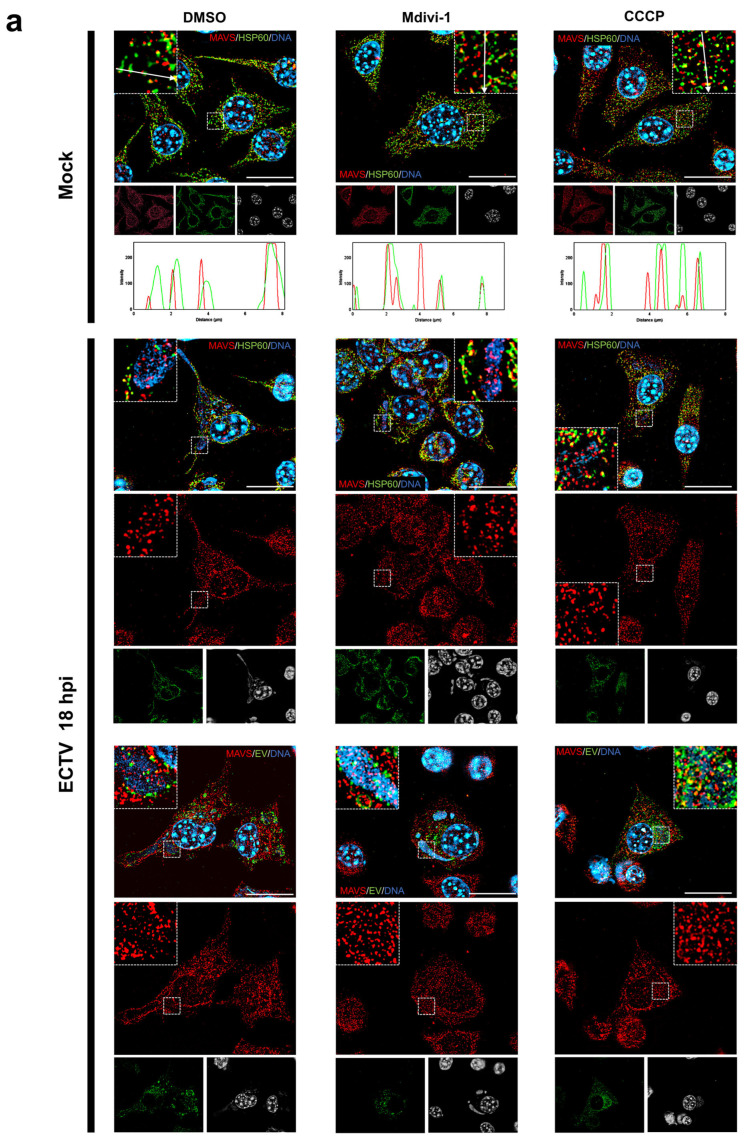

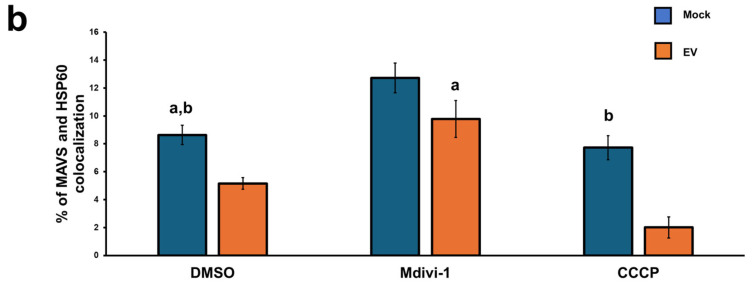
Distribution of MAVS protein in L929 cells at 18 hpi with ECTV. Cells were treated with DMSO, Mdivi-1, or CCCP. (**a**) Magnifications indicate MAVS localization in the viral factories. Arrows show the direction of fluorescence intensity measurements. Fibroblasts are labeled using antibodies against MAVS (red), HSP60 (green), or ECTV (EV; green) and DNA (blue). Scale bar: 20 µm. (**b**) Percentage of MAVS and HSP60 colocalization (*n* = 20). Different letters indicate significant differences (*p* < 0.05) between groups.

The immunofluorescence technique revealed that MAVS protein predominantly localizes to mitochondria in uninfected control L929 cells, irrespective of changes in mitochondrial network morphology. The percentage of MAVS and HSP60 (mitochondrial marker) colocalization between groups is presented in Figure 3b. An elongated mitochondrial network indicates the highest colocalization level (mock: 12.72 ± 1.07%; ECTV: 5.16 ± 0.42%) compared to a normal (mock: 8.63 ± 0.69%; ECTV: 5.16 ± 0.42%) and fragmented (mock: 7.72 ± 0.86%; ECTV: 2.01 ± 0.76%) network both in mock and ECTV-infected cells. However, upon ECTV infection, there is a partial accumulation of this protein in viral factories (Figure 3a). The close proximity of MAVS to the site of virus replication may suggest its involvement in an antiviral signaling pathway during ECTV infection. Conversely, it could also signify an anti-immune viral mechanism, though confirming this necessitates further research. Furthermore, the localization of MAVS outside mitochondria within ECTV factories in L929 cells suggests the possibility of peroxisome localization of MAVS or its release into the cytoplasm. 

**Figure 4:**
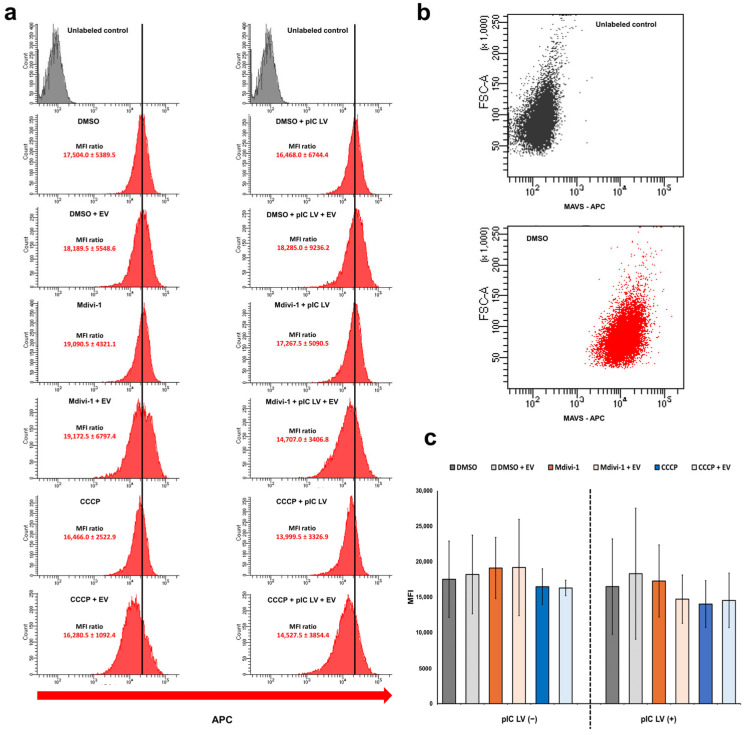
Flow cytometry analysis of MAVS protein level in L929 cells at 24 hpi with ECTV (EV). (**a**) Representative histograms indicate the fluorescence intensity of APC-labeled cells treated with DMSO, Mdivi-1, or CCCP and with or without transfection with poly(I:C)(HMW) LyoVec (pIC LV). A black line is drawn through the center of the histogram of uninfected DMSO-treated cells. (**b**) Dot plots of unlabeled and APC-labeled cells treated with DMSO. (**c**) Mean fluorescence intensity (MFI) of APC in cells treated with DMSO, Mdivi-1, or CCCP and with or without transfection with poly(I:C)(HMW) LyoVec.

**Figure 5:**
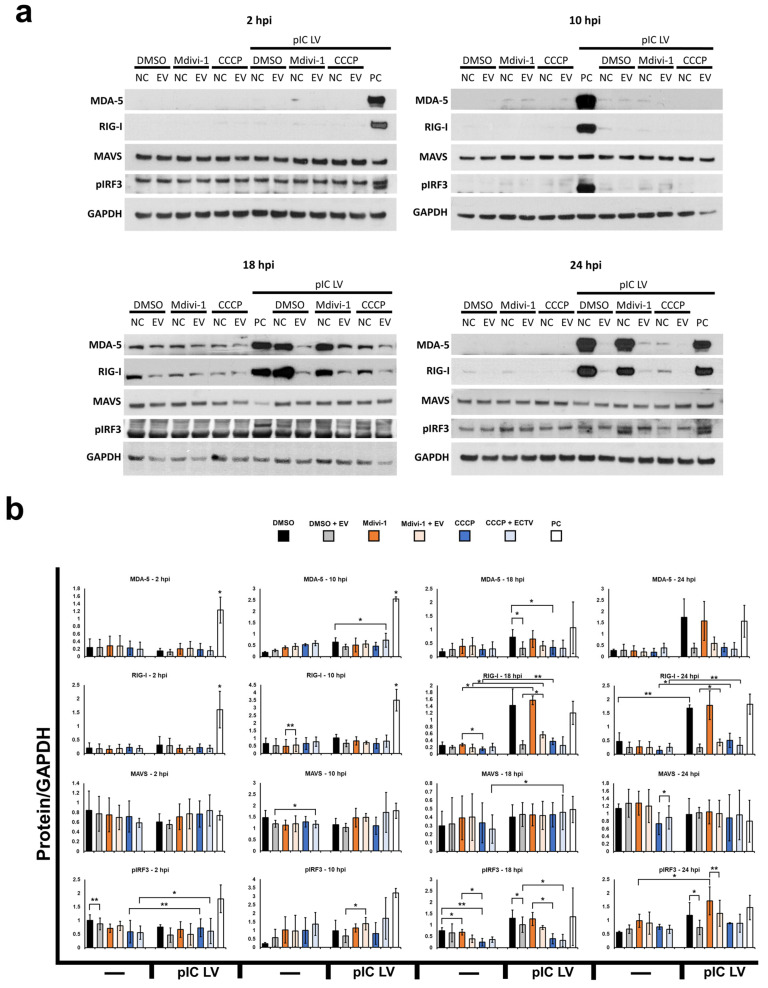
Western blot analysis of proteins involved in MAVS-dependent immunity in L929 cells treated with DMSO, Mdivi-1, or CCCP during ECTV infection. (**a**) Representative Western blots of MDA-5, RIG-I, MAVS, pIRF3, and STING at 2, 10, 18, and 24 h post infection (hpi) with ECTV (EV). (**b**) Densitometry analysis of MDA-5, RIG-I, MAVS, pIRF3, and STING at 2, 10, 18, and 24 hpi with ECTV. The level of each protein was normalized to glyceraldehyde-3-phosphate dehydrogenase (GAPDH). The data ARE presented as mean ± standard deviation (SD), with statistical significance indicated as * *p* ≤ 0.05 AND ** *p* ≤ 0.01. pIC LV—cells transfected with poly(I:C)(HMW) LyoVec.

Both flow cytometry analyses (Figure 4) and Western blot (Figure 5) indicate that MAVS protein levels remain without significant change regardless of mitochondrial network morphology modification during ECTV infection (at 2, 10, 18, and/or 24 hpi) and/or transfected with poly(I:C)(HMW) LyoVec, a RIG-I-like receptor (RLR) agonist. However, it was observed that CCCP treatment could partially decrease the level of MAVS in L929 cells at the late stages of infection (18 and/or 24 hpi).

In conclusion, the results show that neither ECTV infection, alterations in mitochondrial network morphology, nor RLR-agonist stimulation significantly affect the MAVS level in L929 fibroblasts.

**Figure 6:**
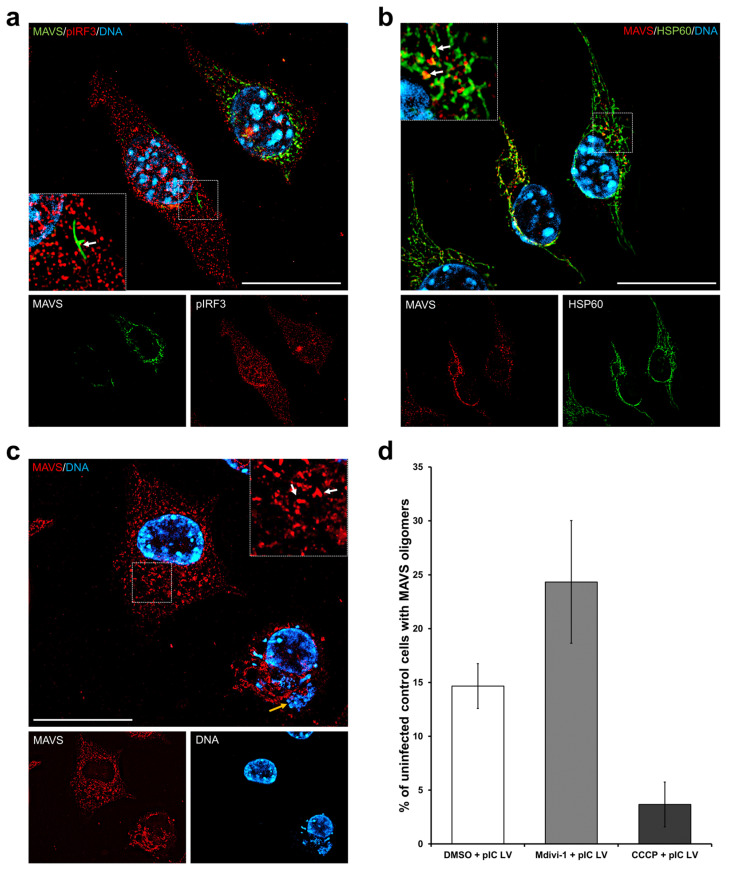
Oligomerization of MAVS protein in L929 cells transfected using poly(I:C)(HMW) LyoVec at 24 hpi with ECTV. (**a**,**b**) Uninfected control cells treated with Mdivi-1. Fibroblasts were labeled with specific antibodies against (**a**) MAVS (green) and pIRF3 (red) or (**b**) MAVS (red) and Hsp60 (green)—marker of mitochondria. (**c**) ECTV-infected cells treated with Mdivi-1 and labeled with specific antibodies against MAVS (red). DNA was labeled with DAPI (blue). White arrows indicate MAVS oligomers, the yellow arrow shows viral factory, and the white arrowhead indicates pIRF3 in the nucleus. Scale bar: 20 µm. (**d**) The bar chart presents the percentage of uninfected control cells with MAVS oligomers. Fibroblasts were treated with DMSO, Mdivi-1, or CCCP.

Oligomerization of MAVS protein and translocation of the transcription factor pIRF3 to the cell nucleus following 24 h poly(I:C)(HMW) LyoVec stimulation in L929 fibroblasts was also observed (Figure 6a,b). These instances signify MAVS protein activation and subsequent induction of MAVS-dependent immune mechanisms, corroborating the existing literature [16,17,18]. The highest number of activated cells was noted in the Mdivi-1-treated samples (24.3 ± 5.7%), while the lowest was observed in the CCCP-treated fibroblasts (3.7 ± 2.1%) (Figure 6d). After treatment with DMSO, approximately 14.7 ± 2.1% of cells exhibited MAVS oligomerization. MAVS oligomerization at specific sites was associated with a decrease in fluorescence signal from this protein across the rest of the mitochondrial network. 

In fibroblasts infected with ECTV (at 24 hpi) and transfected with poly(I:C) LyoVec, MAVS oligomerization and concurrent virus presence within the cell were not observed (Figure 6c). Only a few neighboring fibroblasts adjacent to infected cells exhibited MAVS activation.

### 3.4. Mitochondrial Elongation Reduces the ECTV-Dependent Downregulation of RIG-I-like Receptors Level and IFN-β Concentration in Murine L929 Fibroblast during Late Stages of Infection

RIG-I-like receptors, primarily situated in the cell cytoplasm, play a pivotal role in direct binding to free dsRNA molecules, particularly those of viral origin [3]. The replication process of poxviruses, including ECTV, leads to the formation of dsRNA, which in turn can induce the production of interferons and pro-inflammatory cytokines, contingent upon RLR activation [4]. The Western blot’s results showed that ECTV infection significantly reduces the levels of RIG-I and MDA-5 receptors in L929 cells treated with poly(I:C) LyoVec at 18 and 24 hpi in all three groups, i.e., cells with unaltered mitochondrial network morphology (DMSO), with an elongated network (Mdivi-1), and with a fragmented network (CCCP) (Figure 5). However, infected fibroblasts treated with Mdivi-1 exhibited elevated levels of both RLRs compared to the other two groups. Similar observations were noted regarding the levels of phosphorylated IRF3, indicating activation of RLR. It can be suggested that the fluctuations of RIG-I, MDA-5, or pIRF3 levels are consequences of poxviral E3 protein activity.

**Figure 7:**
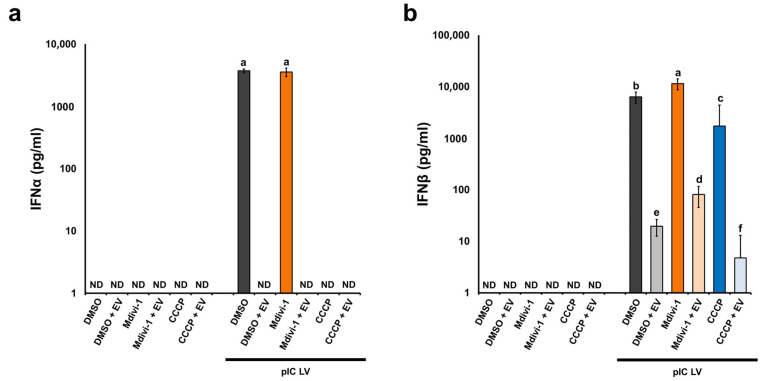
Extracellular level of IFN-α (**a**) and IFN-β (**b**) produced by L929 at 24 hpi with ECTV (EV). Cells were treated using DMSO, Mdivi-1, or CCCP and/or transfected with poly(I:C)(HMW) LyoVec (pIC LV). ND—non-detected. Different letters indicate significant differences (*p* < 0.05) between groups.

The results obtained with the ELISA indicate that cells with an elongated mitochondrial network produced more IFN-β following poly(I:C) LyoVec stimulation compared to cells with unaltered and fragmented networks (Figure 7a). However, no such relationship was observed for IFN-α (Figure 7b). Conversely, mitochondrial network fragmentation significantly or completely inhibited IFN-α and IFN-β production in infected and uninfected L929 cells treated with poly(I:C) LyoVec. Additionally, there was a lack of synthesis of pro-inflammatory cytokines, i.e., IL-6, IL-1β, and TNF-α, in all tested L929 cells groups, both infected and uninfected as well as treated and untreated with poly(I:C) LyoVec.

This may suggest that mouse L929 fibroblasts might be incapable of producing proinflammatory cytokines in response to stimulation with poly(I:C)(HMW) LyoVec. Another hypothesis could be attributed to the potential insensitivity of the ELISAs employed for detecting IL-6, IL-1β, and TNF-α in these cells.

### 3.5. Elongation of the Mitochondrial Network Increases the Degree of Colocalization of MAVS with RIG-I-like Receptors and STING in Uninfected L929 Fibroblasts

**Figure 8:**
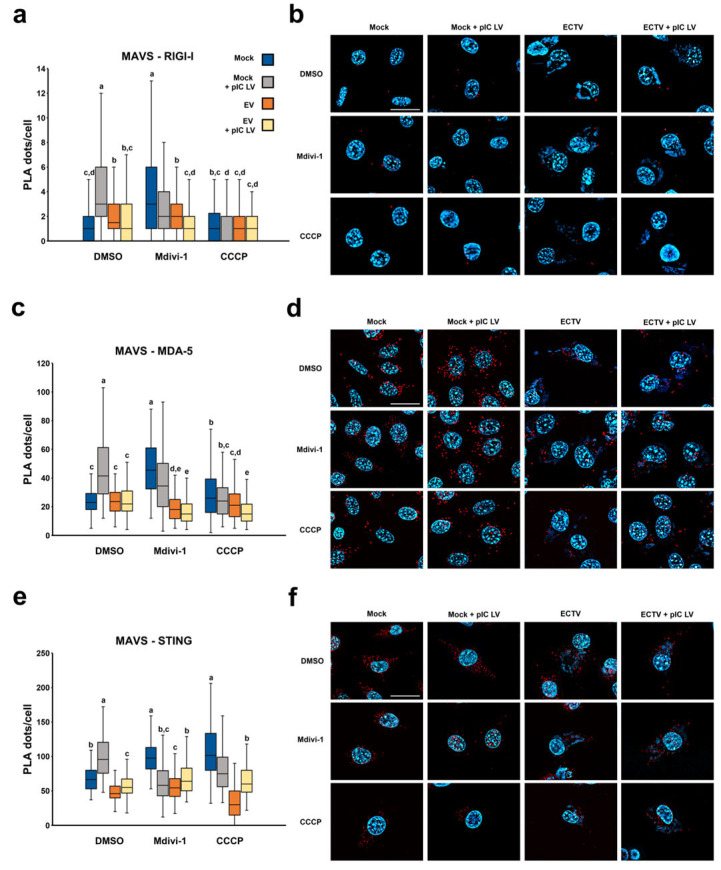
Colocalization of MAVS protein with RIG-I (**a**,**b**), MDA-5 (**c**,**d**), and STING (**e**,**f**) in L929 cells at 18 hpi with ECTV. Cells were treated using DMSO, Mdivi-1, or CCCP and/or transfected with poly(I:C)(HMW) LyoVec. Box plots (**a**,**c**,**e**) indicate the number of PLA dots per cell. Different letters indicate significant differences between groups (*p* ≤ 0.05). (**b**,**d**,**f**) Representative figures visualize the number of PLA dots per cell in each group. Scale bar: 20 µm.

Visualization of MAVS in close vicinity with RIG-I, MDA-5, or STING was performed using proximity ligation assay. Mdivi-1 treatment, which results in an elongated mitochondrial network, increases the degree of colocalization of MAVS protein with cytoplasmic RIG-I and MDA-5 receptors as well as with STING protein on the endoplasmic reticulum relative to the unaltered network in uninfected L929 cells (Figure 8). In contrast, ECTV infection (18 hpi) was found to nullify this effect and cause a reduction in the degree of MAVS–STING, MAVS–MDA-5, and MAVS–RIG-I colocalization in L929 cells relative to uninfected cells in most of the groups studied. 

The above results indicate that ECTV infection blocks the interaction of proteins involved in signal transduction to produce type I IFN and pro-inflammatory cytokines, thereby inhibiting the antiviral response.

In the case of MAVS–STING colocalization, this may be related to the disorganization of the mitochondrial network in these cells found in previous studies [7], which probably also affects the interaction with the endoplasmic reticulum and thus with the STING protein. In contrast, the reduction in colocalization between MAVS and RIG-I as well as MDA-5 during ECTV infection may stem from a significant decline in the cellular levels of these receptors, which was previously detailed in Section 3.4.

### 3.6. ECTV Infection and Modifications of Mitochondrial Network Morphology Affect Colocalization of MAVS with Fusion/Fission Proteins

**Figure 9:**
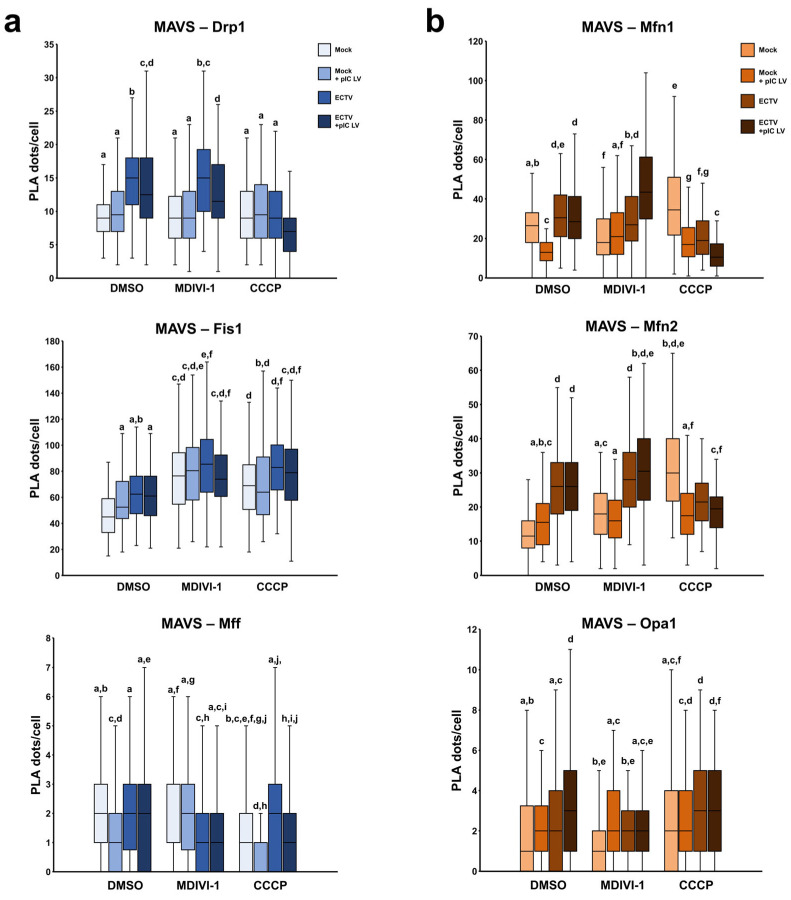
Colocalization of MAVS with fission (**a**) and fusion (**b**) proteins in L929 at 18 hpi with ECTV. Cells were treated using DMSO, Mdivi-1, or CCCP and/or transfected with poly(I:C)(HMW) LyoVec. Box plots indicate the number of PLA dots per cell. Different letters indicate significant differences between groups (*p* ≤ 0.05).

The proximity ligation assay was utilized to visualize MAVS along with one of the following: (1) Drp1, Fis1, and Mff, i.e., proteins associated with mitochondrial fission (Figure 9a), or (2) Mfn1, Mfn2, and Opa1, i.e., proteins linked to mitochondrial fusion (Figure 9b). The highest number of PLA dots was observed during the examination of MAVS–Fis1 colocalization, with a median value between 45.0 (DMSO) and 85.5 (Mdivi-1 + poly(I:C)(HMW) LyoVec) per cell. However, the lowest number was observed in the case of MAVS–Mff colocalization, with a median value between 1.11 (CCCP + poly(I:C)(HMW) LyoVec) and 2.17 per cell. The presented results indicate that the elevated occurrence of MAVS–Fis1 colocalization might be related to the abundant presence of Fis1 on the mitochondrial outer membrane (MOM), facilitating frequent encounters of these proteins. In contrast, while Mff also functions as a mitochondrial fission protein and is localized on the MOM, the significantly lower MAVS–Mff colocalization implies a lower Mff concentration than Fis1 or infrequent proximity with MAVS. Despite statistically significant differences between groups, a deficient number of MAVS–Mff PLA dots per cell makes a fair comparison difficult. Similar considerations apply to the colocalization assessment of MAVS and Opa1; however, given that Opa1 is primarily located on the mitochondrial inner membrane (MIM), the prospect of MAVS and Opa1 interaction may be diminished.

Our study showed that ECTV infection increases MAVS–protein colocalization in nearly all cases in L929 cells with either an unaltered or elongated mitochondrial network. Surprisingly, ECTV infection did not alter MAVS–Drp1 and MAVS–Fis1 colocalization in cells with a fragmented mitochondrial network. The results suggest that mitochondrial fragmentation inhibits the increased impact of ECTV infection on the MAVS colocalization with fission proteins. 

Moreover, compared to uninfected controls, the proximity of MAVS–Mfn1 and MAVS–Mfn2 was notably decreased during infection. In addition, CCCP treatment increases MAVS–Mfn1 and MAVS–Mfn2 colocalization in uninfected controls in comparison to cells with physiological mitochondrial network morphology, which could indicate a potential role for mitochondrial dynamics in regulating MAVS-mediated antiviral signaling pathways.

## 4. Discussion

Our previous studies have shown that ECTV infection induces mitochondrial network disorganization and fragmentation in murine fibroblasts and macrophages [14,15]. Building on this observation, we sought to investigate how modifications in mitochondrial network morphology affect MAVS-dependent antiviral immunity. We opted to utilize chemicals such as Mdivi-1 and CCCP to alter mitochondrial network morphology rather than employing gene silencing techniques. This decision was influenced by the potential therapeutic implications of Mdivi-1, a mitochondrial division inhibitor, which holds promise in treating neurodegenerative diseases like Parkinson’s, Alzheimer’s, and multiple sclerosis or some others like atherosclerosis [19,20]. Furthermore, studies on the coxsackie B virus have demonstrated that Mdivi-1 treatment diminishes the extracellular virus titer in infected cells, indicating the potent antiviral effectiveness of this drug [21]. Comparable outcomes have been observed in cells infected with SARS-CoV-2, rotavirus, and Venezuelan equine encephalitis virus (VEEV) [22,23,24]. Additionally, Shukla and colleagues (2022) found that the combined administration of Mdivi-1 with subunit vaccines against SARS-CoV-2 and inactivated vaccines targeting H1N1 influenza notably enhanced vaccine effectiveness and provided increased protection against lethal H1N1 viral challenge [25]. Yang and colleagues (2020) demonstrated that Mdivi-1’s inhibition of mitochondrial fragmentation induced by Zika virus (ZIKV) infection leads to enhanced cell survival [26]. Thus, reinstating the balance between mitochondrial fission and fusion could serve as a strategy to alleviate the cellular damage caused by ZIKV infection. The present study showed that an elongated mitochondrial network enhances MAVS-dependent immunity during ECTV infection, as manifested by an increased level of IFN-β and decreased virus titer, implying that Mdivi-1 holds promise as a prospective antipoxviral therapy. The following research stage should involve assessing the antipoxviral effects of the drug Mdivi-1 through in vivo studies. 

The research findings demonstrated that modifications in the morphology of the mitochondrial network impact MAVS-dependent immunity in L929 murine fibroblasts infected with the ECTV. However, it was observed that ECTV infection significantly inhibits the activation of RLRs, MAVS, and the phosphorylation of IRF3 in this cell line unless supplemented with additional stimulation using poly(I:C)(HMW) LyoVec. These results contradict the findings of Cheng and colleagues (2018), where L929 cells exhibited pIRF3 formation and, subsequently, IFN-β production upon ECTV infection itself [27]. The observed variations may arise from using different strains of the ECTV. ECTV-MOS, employed in the current study, is recognized as the most virulent among mousepox virus strains [28,29]. Conversely, its wild-type counterpart is expected to be less pathogenic, as corroborated by Cheng and colleagues (2017) in a separate publication [30].

Our earlier studies revealed significant alterations in the distribution of mitochondria during ECTV infection [14,15]. Specifically, mitochondria tend to accumulate near the cell nucleus or between the nucleus and the viral factory, potentially indicating a strategic adaptation to enhance ATP production conducive to viral replication. This observed phenomenon suggests a potential association between the repositioned mitochondria and viral energy requirements. 

Our present studies indicated that MAVS predominantly localizes to mitochondria in uninfected L929 murine fibroblasts, although a fraction of this protein is distributed outside the mitochondria. Numerous studies have demonstrated the presence of MAVS on peroxisomes, small eukaryotic organelles involved in various metabolic processes such as fatty acid oxidation and detoxification [31,32,33]. Intriguingly, in ECTV-infected cells, the extra-mitochondrial MAVS protein was found in proximity to viral factories, suggesting a potential role in antiviral immunity (peroxisomal MAVS) or virus evasion mechanisms, which requires further research.

**Figure 10:**
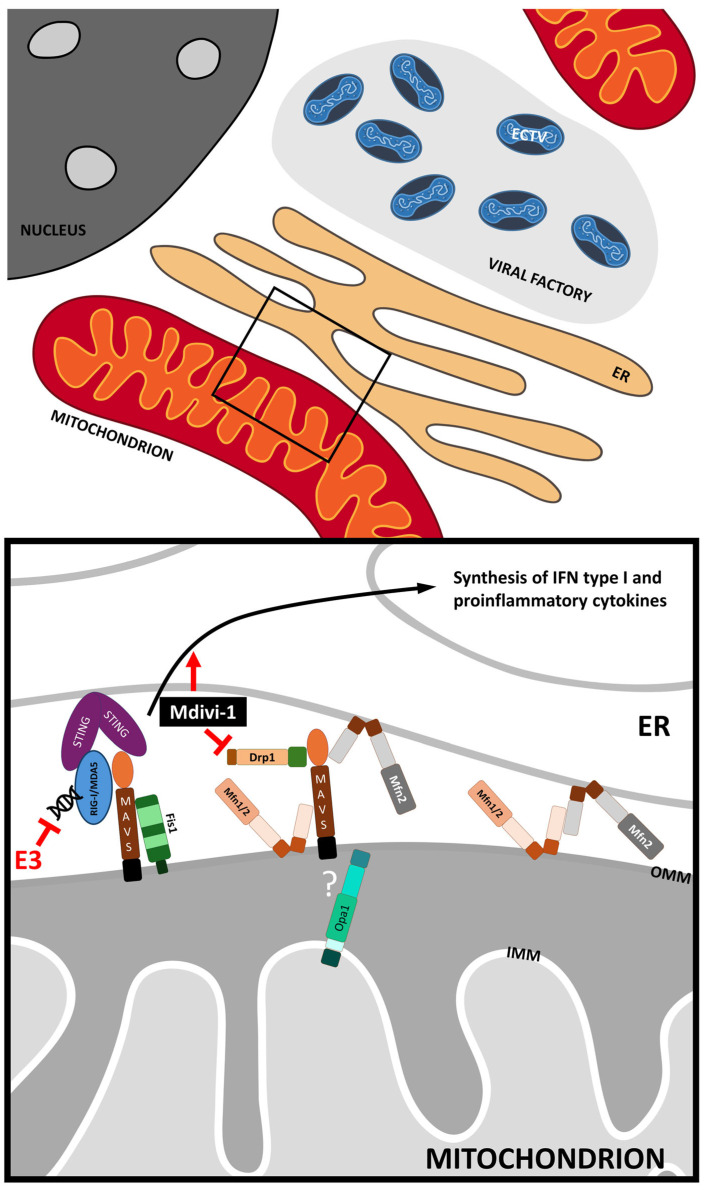
Possible interaction between MAVS and selected proteins. E3—poxviral protein; ER—endoplasmic reticulum; Drp1—dynamin-related protein 1; Fis1—Fission 1 protein; IMM—inner mitochondrial membrane; MAVS—mitochondrial antiviral signaling protein; MDA5—melanoma differentiation-associated protein 5; Mdivi-1—mitochondrial division inhibitor; Mfn1/2—mitofusin 1/2; OMM—outer mitochondrial membrane; Opa1—optic atrophy 1; RIG-I—retinoic acid-inducible gene I; STING—stimulator of interferon genes.

Since our study involved modifying the mitochondrial network through fragmentation and elongation, we decided to investigate the interaction of MAVS with proteins involved in mitochondrial fusion and fission. Our study indicated an increased colocalization of MAVS with Mfn1/2 during infection only in cells exhibiting an unaltered or elongated mitochondrial network, which could imply enhanced interactions between these proteins. However, a significantly lower number of MAVS–Mfn1/2 PLA dots in cells with fragmented mitochondria than those with unaltered or elongated organelles was noted. This finding strongly indicates that mitochondrial fission considerably reduces the possibility of contact between these mentioned proteins. Several studies have shown an interplay between MAVS and both mitofusins, albeit with varying consequences on MAVS activity [10,34,35]. It was reported that Mfn1 acts as a positive regulator of MAVS-mediated antiviral signaling; however, Mfn2 effectively impedes MAVS function by forming a robust interaction with it. Interestingly, our results revealed strikingly similar alterations in the number of PLA dots representing both MAVS–Mfn1 and MAVS–Mfn2 colocalization. Consequently, the precise nature of these interactions—whether they are anti- or provirus—during ECTV infection remains unclear. Nevertheless, the enhanced MAVS–Mfn1/2 colocalization in the unaltered or elongated mitochondrial network may arise from intensified contact between the membranes of perinuclear clustered mitochondria in the infected cells. This facilitates more frequent encounters of MAVS and mitofusins. Mitochondrial fragmentation may exert the opposite effect. Furthermore, the perinuclear region is characteristic of the endoplasmic reticulum (ER) localization [36,37,38], where mitochondrial accumulation occurs in the L929 cells infected with ECTV [15]. It suggests the formation of mitochondria-associated ER membranes (MAMs) in this vicinity, which may facilitate the assembly of viral particles. The close proximity between mitochondria and ER is modulated by the interaction of mitofusins, proteins found on both organelles (Figure 10). This highlights their role in enabling direct contact between these compartments [39,40,41]. Additionally, it may suggest that MAMs play a crucial role in virus replication and assembly.

However, our investigation also indicated increased MAVS–STING colocalization in cells with elongated mitochondrial network. STING protein localizes on ER and plays a crucial role in an anti-DNA virus response. The activation pathways of MAVS and STING intersect at the level of TBK1 (TANK-binding kinase 1) and proceed along the same pathway from that point forward [42]. Castanier et al. (2010) suggested that the branchy mitochondrial network increases the contact between mitochondria and ER, allowing for a greater number of MAVS-STING interactions [10]. Then, the production of IFN type I and proinflammatory cytokines is intensified. On the other hand, they found that the extensive mitochondrial fragmentation inhibits the MAVS-dependent signaling pathway, which was confirmed in our current studies. Nevertheless, contrary to expectation, we observed an increased level of MAVS–STING colocalization in fibroblasts with fragmented mitochondria compared to cells with unaltered organelles. An explanation for this statement might be cellular stress triggered by treatment with CCCP [43]. Its toxicity in L929 fibroblast was observed at an approximately 20% level. It could have been enough to release mitochondrial DNA to the cytoplasm and induce the cGAS–STING antivirus pathway to produce IFN type I and proinflammatory cytokine. However, Kwon and colleagues (2017) indicated that CCCP suppresses the STING-mediated DNA-sensing pathway [44]. Confirming or denying these results on L929 cells requires further, more detailed studies. 

Nevertheless, our findings revealed that infection with ECTV results in decreased levels not only of MAVS–STING but also MAVS–RIG-I and MAVS–MDA-5 colocalization in L929 cells, irrespective of mitochondrial network morphology. This strongly implies that ECTV infection disrupts the interaction between proteins crucial for initiating antiviral signaling pathways dependent on MAVS and STING. It is unknown if the ECTV encodes specific protein/s responsible for the destruction of protein–protein interaction or for binding individual RIG-I, MDA-5, MAVS, and/or STING. However, Chang et al. (1992) indicated that the E3 protein (encoded by the E3L gene) of VACV binds to double-stranded RNA (dsRNA) using its C-terminal dsRNA-binding domain, thereby preventing the activation of RLR [45]. The E3L gene is conserved across most members of *Chordopoxvirinae* [46,47,48], including ECTV; thus, inhibition of RLR signaling was observed in our studies. This might indicate that ECTV E3 protein indirectly reduces MAVS–RIG-I and MAVS–MDA-5 colocalization. However, disruption of protein–protein interaction may be related also to virus-mediated cellular changes. Subsequent research should focus on the mechanism/s involved in these processes. 

Moreover, we confirmed the findings of other researchers that colocalization of MAVS and Drp1 proteins indeed occurs [49,50]. Drp1 is a cytoplasmic protein responsible for mitochondrial fission [51,52,53]. It recognizes and binds to the specific receptors on the mitochondria, such as Fis1 and Mff. Chen and colleagues (2020) revealed that MAVS and Drp1 not only interact with each other but also have a strong affinity when detecting RNA [50], which can appear in the cytosol during viral infection. Our current work indicated that colocalization of MAVS and Drp1 was elevated in the course of ECTV infection in L929 cells with normal and elongated mitochondrial networks. It is likely associated with the higher concentration of Drp1 in the ECTV-infected fibroblasts’ cytoplasm, as shown in our previous studies [14]. Furthermore, the initiation step of mitochondrial fragmentation causes the recruitment of such protein to mitochondria, explaining the increased chance of its meeting with MAVS. Surprisingly, our work indicates that using Drp1 inhibitor–Mdivi-1 does not alter the colocalization level of MAVS and Drp1 compared to cells with the physiological mitochondrial network. This implies that while Mdivi-1 inhibits the activation/phosphorylation of Drp1, the distribution of this protein to the mitochondria and its concentration likely remain unchanged. Moreover, our study indicated colocalization between Fis1 and MAVS, with a higher level in L929 fibroblasts displaying the elongated and fragmented mitochondrial network than in a physiological case condition. Fis1 and MAVS are located on the mitochondrial outer membrane, which should promote their contact. However, the increasing impact of ECTV infection appears notable only in DMSO-treated cells. This does not seem surprising since our previous work indicated that the level of Fis1 elevated at the late stages of infection with ECTV in L929 cells, providing the opportunity for close contact between MAVS and Fis1 [14]. Despite the minimal higher level of MAVS-Fis1 colocalization in Mdivi-1-treated cells and insignificant changes in CCCP-treated cells during ECTV infection, mitochondrial network modifications increase the opportunity for MAVS and Fis1 colocalization in both ECTV-infected and noninfected-control murine fibroblasts. This may be caused by the higher possibility of contact between MAVS and Fis1 on elongated mitochondria due to the greater membrane surface area. However, increased colocalization in cells with fragmented mitochondria suggests that Fis1 concentration should probably be higher than in DMSO- or Mdivi-1-treated cells, which would promote protein contact. Nevertheless, many studies have shown that mitochondrial fission decreases MAVS signaling [54,55,56,57,58]. Considering the above, the role of Fis1 or Drp1 in enhancing or reducing MAVS signaling still remains unclear and requires future studies. 

## 5. Conclusions

Our study demonstrated that modifications of mitochondrial network morphology affect MAVS-dependent immunity in ECTV-infected mouse L929 fibroblasts. Elongation of mitochondria by treatment with Mdivi-1 reduces the number of progeny virions and increases the level of IFN-β, offering the potential for future use as an antipoxviral/antiviral drug.

## Figures and Tables

**Table 1:** Primary antibodies.

Antibody	Isotype	Company	Protein Function
MAVS	Mouse monoclonal	Santa Cruz Biotechnology Dallas, TX, USA	A mitochondrial platform for signal transduction to production of IFN type I and proinflammatory cytokines
MAVS	Rabbit polyclonal	Thermo Fisher Scientific Waltham, MA, USA	As above
STING	Rabbit polyclonal	Thermo Fisher Scientific Waltham, MA, USA	An endoplasmic reticulum platform for signal transduction to production of IFN type I and proinflammatory cytokines
RIG-I	Rabbit polyclonal	Thermo Fisher Scientific Waltham, MA, USA	A cytosolic pattern recognition receptor
MDA-5	Rabbit polyclonal	Thermo Fisher Scientific Waltham, MA, USA	A cytosolic pattern recognition receptor
pIRF3	Rabbit polyclonal	Thermo Fisher Scientific Waltham, MA, USA	A transcription factor
Drp1	Mouse monoclonal	BD Biosciences San Jose, CA, USA	Cytoplasmic protein required for mitochondrial fission
Mff	Mouse monoclonal	Santa Cruz Biotechnology Dallas, TX, USA	Mitochondrial fission protein
Fis1	Rabbit polyclonal	Thermo Fisher Scientific Waltham, MA, USA	Mitochondrial fission protein
Mfn1	Mouse monoclonal	Abcam Cambridge, UK	Mitochondrial fusion protein
Mfn2	Mouse monoclonal	Abcam Cambridge, UK	Mitochondrial fusion protein
Opa1	Mouse monoclonal	BD Biosciences San Jose, CA, USA	Mitochondrial fusion protein
HSP60	Mouse monoclonal	BD Biosciences San Jose, CA, USA	Heat shock protein–mitochondrial marker
ECTV-FITC	Rabbit polyclonal	–	ECTV antigens
GAPDH	Mouse polyclonal	Thermo Fisher Scientific Waltham, MA, USA	A multifunction constitutively expressed enzyme-loading control in Western blot

## Data Availability

The detailed data for this study are available from the corresponding author upon request.
